# A comparison of clinico-pathologic characteristics of patients with serous and clear cell carcinoma of the uterus

**DOI:** 10.4274/tjod.14478

**Published:** 2016-09-15

**Authors:** Işın Üreyen, Alper Karalok, Derya Akdağ Cırık, Tolga Taşçı, Osman Türkmen, Günsü Kimyon Cömert, Nurettin Boran, Gökhan Tulunay, Taner Turan

**Affiliations:** 1 Antalya Training and Research Hospital, Clinic of Gynecology and Obstetrics, Divisin of Gynecological Oncology, Antalya, Turkey; 2 Etlik Zübeyde Hanım Women’s Health Training and Research Hospital, Clinic of Gynecologic Oncology, Ankara, Turkey

**Keywords:** Uterine serous carcinoma, uterine clear cell carcinoma, Survival, recurrence

## Abstract

**Objective:**

Serous carcinoma and clear cell carcinomas account for 10% and 3% of endometrial cancers but are responsible for 39% and 8% of cancer deaths, respectively. In this study, we aimed to compare serous carcinoma and clear cell carcinoma regarding the surgico-pathologic and clinical characteristics, and survival, and to detect factors that affected recurrence and survival.

**Materials and Methods:**

We retrospectively analyzed patients with clear cell and serous endometrial cancer who underwent surgery between January 1993 and December 2013 in our clinic. We used Kaplan-Meier estimator to analyze survival.

**Results:**

The tumor type in 49 patients was clear cell carcinomas and was serous uterine carcinoma in 51 patients. Advanced stage (stage III and IV) disease was present in 42% of the patients in the clear cell group, whereas this rate was 62% in the serous group (p=0.044). Lymph node metastasis was detected in 37% of the patients with clear cell carcinomas and 51% of the patients with serous carcinoma (p=0.17). The adjuvant therapies used did not differ significantly between the groups (p=0.192). The groups had similar recurrence patterns. Five-year progression-free survival and the 5-year overall survival were 60.6% and 85.8%, 45.5% and 67.8% in the patients with clear cell carcinomas and serous tumor, respectively.

**Conclusion:**

With the exception that more advanced stages were observed in patients with serous carcinoma endometrial cancers at presentation, the surgico-pathologic features, recurrence rates and patterns, and survival rates did not differ significantly between the groups with clear cell carcinoma and serous carcinoma endometrial cancers.

## INTRODUCTION

Endometrial cancer (EC) is the sixth most common cancer in women worldwide and the twelfth most common cancer overall^(1)^. Bokhman^(2)^ first suggested that there were 2 types of ECs that had different microscopic, clinical, epidemiologic, and genetic properties in 1983. Type I ECs, represented by endometrioid carcinomas, constitute 80% of all ECs. Type II tumors are composed of other cell types of ECs that are less frequently observed. Most common cell types included in type II ECs are serous carcinoma (SC) and clear cell carcinoma (CCC). Type II tumors are estrogen independent, seen in older and thinner patients, diagnosed at more advanced stages, and the likelihood of recurrence and death of the disease is much higher with type II compared with type I tumors^(3)^.

SC and CCCs account for 10% and 3% of ECs but are responsible for 39% and 8% of cancer deaths, respectively^(4)^. Therefore, it is important to approach these tumors differently and to separate them from endometrioid tumors in studies that analyze patients with ECs. They are usually analyzed together in studies that investigate characteristic features, management, and survival of patients with these tumors they are rare are more aggressive than endometrioid ECs. This may also explain the disparate results obtained in the literature, because these two tumors are also different with distinct clinical behavior and pathogenetic properties. They have different molecular alterations and separate ways of spreading. Most serous tumors have p53 mutation because only 14% of clear cell tumors have this mutation. Serous tumors have a tendency to spread intraperitoneally just like its ovarian counterparts, whereas clear cell tumors show a propensity for distant metastasis^(5)^. Many studies have compared these tumors with type I or poorly-differentiated ECs. However, there is little data in the literature with regard to the differences between these 2 tumors.

In this study, we aimed to present a comparison of SC and CCC in terms of surgico-pathologic and clinical features, and survival, and to determine factors that affect recurrence and survival.

## MATERIALS AND METHODS

Patients with clear cell and serous EC who underwent surgery in our clinic between January 1993 and December 2013 were retrospectively analyzed. The data related to demographic characteristics, intraoperative findings, surgico-pathologic results, patients’ treatments, recurrence and the site of recurrence, and survival were collected from the electronic gynecologic oncology clinic database system, pathology reports, and surgical records. Patients were staged according to the 2009 International Federation of Gynecology and Obstetrics criteria. Institutional review board approval was obtained.

In our clinic, patients with a preoperative or intraoperative pathologic diagnosis of clear cell or serous tumor undergo staging surgery directly. Staging surgery involves total abdominal hysterectomy, bilateral salpingo-oophorectomy, systematic pelvic and paraaortic lymphadenectomy, abdominal cytology, and omentectomy as standard. Omentectomy was performed as total, infracolic or omental biopsy according to the cell type, intraoperative examination, and decision of the surgeon. Cytoreductive surgery was performed in addition to staging surgery in case there was macroscopic disease intraoperatively.

In terms of adjuvant therapy, only radiotherapy or sandwich therapy (3 cycles of chemotherapy followed by radiotherapy, and then 3 cycles of chemotherapy) or only chemotherapy or radiotherapy followed by chemotherapy were applied at the discretion of the surgeon and according to the stage of the disease.

Patients were followed-up every 3 months in the first 2 years after adjuvant therapy, within every six months until the fifth year, and yearly thereafter. Pelvic examination, complete blood count and blood chemistry, and abdominal ultrasonography were performed at every follow-up. Chest X-ray was performed yearly or in the event of clinical suspicion. Thoracic and/or abdominal computerized tomography was used when needed. In the follow-up, a Papanicolaou smear and cancer antigen-125 (Ca) level tests were used, even though we did not use them routinely.

Pelvic recurrence was defined as recurrence distal to the pelvic inlet (true pelvis), upper abdominal recurrence as recurrence between the pelvic inlet and the diaphragm, and extraabdominal recurrence as all other recurrences. Ascites and peritonitis carcinomatosa were included in the upper abdominal recurrences, whereas recurrence in the liver parenchyma and bone were included in the extraabdominal recurrences. The period from surgery to recurrence or last visit was defined as progression-free survival (PFS), and the period from surgery to death or last visit was defined as overall survival (OS). Follow-up time was evaluated as the time between surgery and the time of the patient’s last examination (death or last visit).

### Statistical Analysis

Statistical data were analyzed using Statistical Package for Social Sciences (SPSS) version 17 (SPSS Inc., Chicago IL, USA). Descriptive statistics are presented using median and range or mean and standard deviation where appropriate. Student’s t-test and the Mann-Whitney U test were used to compare means and medians, respectively, in different groups. The chi-square test or Fisher’s exact test were used where appropriate to compare proportions and percentages in different groups. Kaplan-Meier estimation was used for the analysis of survival. The logrank test and univariate Cox regression analysis were used for analysis of categorical and continuous variables that affect survival, respectively. The possible factors identified with univariate analysis were entered into multivariate Cox regression analysis. Statistical significance was considered as p<0.05.

## RESULTS

### Surgico-pathologic factors

In our clinic, 1640 patients with EC underwent surgery between January 1993 and December 2013. In the defined time period, 100 (6.1%) patients were diagnosed as having either clear cell or serous uterine tumor. Among these, 49 patients had clear cell and 51 patients had serous uterine carcinoma. The mean age at diagnosis was 63±8.2 years. All but seven patients were staged surgically. Forty-two patients had stage 1 disease, five patients had stage 2, 32 patients had stage 3 and 19 patients had stage 4 disease. Forty-two percent of patients had advanced stage (stage III and IV) disease in the clear cell group, and 62% had advanced stage disease in the serous group (p=0.044).

The median number of harvested lymph nodes was 50 (range, 2-118), and these numbers were 16 (range, 1-55) and 37 (range, 2-69) for paraaortic and pelvic regions, respectively. Among the patients with lymphadenectomy, 41 (44%) patients had lymph node metastasis. Of these patients, 16 patients had only pelvic involvement, 8 patients had only paraaortic involvement, and 16 patients had both pelvic and paraaortic involvement. In one patient the region of involvement was not defined. Thirty-seven percent of patients with CCC and 51% of patients with SC had lymph node metastasis (p=0.17). Lymphovascular space invasion (LVSI) was positive in 40 patients; it was positive in 40% and 61% of patients with CCC and serous tumor, respectively (p=0.07). Abdominal cytology was positive in 23 patients. Omental metastasis was observed in 21 patients. Twenty-one (22%) patients had ovarian involvement. The median Ca-125 level was 28 (range, 1-915).

There was no difference between the 2 tumor types in terms of number of harvested lymph nodes, myometrial invasion, cervical involvement, cytologic positivity, tumor size, ovarian involvement, omental metastasis, preoperative Ca-125 level, and the site of recurrence (abdominal vs. extraabdominal) (Table 1).

### Adjuvant therapy

Nine patients had no adjuvant therapy, and 71 patients completed adjuvant therapy. Twenty-one patients had radiotherapy only, 34 patients had chemotherapy only, 11 patients received sandwich therapy, and 5 patients had radiotherapy followed by chemotherapy. Platin- based chemotherapy was used (paclitaxel + carboplatin, n=31; paclitaxel + cisplatin, n=9; cisplatin, n=3; paclitaxel, n=1, doxorubicin + cisplatin, n=5; paclitaxel + carboplatin + epirubicin, n=1). Thirty-five patients completed six cycles of chemotherapy. Eighteen were lost to follow-up following surgery. Eighty percent of patients with CCC and 65% of patients with serous tumor received adjuvant therapy (p=0.08). Details of the adjuvant therapy in patients with CCC and serous tumor are defined separately in Table 2. Radiotherapy seemed to be used more commonly in patients with CCC; however, there was no statistical difference between the groups regarding the type of adjuvant therapies used (p=0.192).

### Recurrence

Recurrence was observed in 22 patients (27.5%). This number was 9 (24%) and 13 (31%) for patients with CCC and SC, respectively (p=0.47). The mean time to recurrence was 12 months (range, 1-45 months). The time to recurrence and site of recurrence were similar in the two tumor types. Recurrence was only in the upper abdomen in nine patients, only extraabdominal in four patients, only in the pelvis in two patients, in the upper abdomen and extraabdominal in three patients, in the pelvis and extraabdominal in two patients, in the pelvis and upper abdomen in one patient, and in the pelvis, upper abdomen, and extraabdominal in one patient. The recurrence pattern of the 2 tumor types is defined in detail in Table 3.

In the subgroup analysis of patients with clear cell tumors, only lymph node involvement was associated with recurrence (p=0.04). Depth of myometrial invasion, LVSI, ovarian involvement, omental metastasis, cervical invasion, age, number of lymph nodes harvested, tumor size, and taking adjuvant therapy were not associated with recurrence. The patients who had recurrence had higher preoperative Ca-125 levels (p=0.014).

In the group with serous tumor, paraaortic lymph node metastasis, presence of LVSI, ovarian and omental metastasis, and higher preoperative Ca-125 level were associated with a higher risk of recurrence (p=0.017, p=0.004, p=0.007, p=0.001 and p=0.007, respectively). On the other hand, pelvic lymph node metastasis, myometrial invasion, positivity of peritoneal cytology, cervical invasion, age, number of lymph nodes harvested, tumor size, and taking adjuvant therapy were not associated with recurrence.

### Survival Analysis

The median follow-up time was 18.5 months (range 1-156 months), and was 39 months (range, 1-156 months) and 28 months (1-96 months) for patients with CCC and SC ECs, respectively (p=0.035). Five patients with CCC and eight patients with SC ECs died in the follow-up period; one patient with CCC died during adjuvant therapy and the other who had SC died before the adjuvant therapy (1 following 3 cycles of chemotherapy and 1 before adjuvant therapy-renal insufficiency).

**Figure 1:**
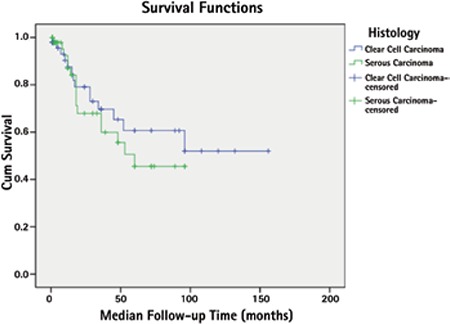
Five-year progression-free survival

**Figure 2:**
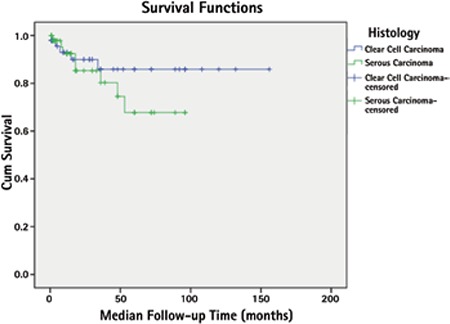
Five-year overall survival

Five-year PFS was 53.6%; 60.6% and 45.5% for CCC and serous tumor, respectively (p=0.465) (Figure 1). Five-year OS was 77.1%; 85.8% and 67.8% for CCC and serous tumors, respectively (p=0.565) (Figure 2).

In the univariate analysis of the clear cell subgroup, omental metastasis, paraaortic involvement, peritoneal cytology positivity, and taking no adjuvant therapy were associated with worse 5-year OS (p=0.002, p=0.003, p<0.001, p=0.035 respectively), and omental metastasis, paraaortic involvement, pelvic involvement, positivity of peritoneal cytology, preoperative Ca-125 level and ovarian metastasis were associated with 5-year PFS (p<0.001, p=0.014, p=0.044, p<0.001, p=0.016 and p=0.006, respectively) (Tables 4 and 5). Multivariate analysis could not be performed in this subgroup.

In the univariate analysis of the serous subgroup, lower number of harvested lymph nodes, paraaortic metastasis, and LVSI were associated with worse 5-year OS (p=0.017, p<0.001, p=0.041 respectively), and paraaortic metastasis, omental metastasis, and LVSI were associated with worse 5-year PFS (p<0.001, p=0.004, p=0.006, respectively) (Tables 4 and 5). Multivariate analysis could not be performed for OS and it could not detect any independent prognostic factor for 5-year PFS.

## DISCUSSION

Type II ECs differ from endometrioid tumors with their less favorable characteristics. They have a tendency to present at more advanced stages and to recur earlier^(6,7)^. In the present study, 42% of patients had advanced stage (stage III and IV) disease in the clear cell group, and 62% had advanced stage disease in the serous group (p=0.044). The rate of advanced stage disease is reported between 40% to 56% in the literature for both serous and clear cell tumors^(3,4,6,7)^. The different rates may be explained by the different rates of comprehensive surgical staging in these studies. SC, ECs seem to present at more advanced stages than CCCs according to our results.

There was no difference between the 2 tumor types in terms of number of harvested lymph nodes, myometrial invasion, cervical involvement, cytologic positivity, tumor size, ovarian involvement, omental metastasis, preoperative Ca-125 level, and the site of recurrence (abdominal vs. extraabdominal). LVSI was positive in 40% and 61% of patients with CCC and serous tumor, respectively (p=0.07). This difference seems clinically significant. However, it did not reach statistical significance. Omental metastasis was observed in 24% of the patients. Although omentectomy is controversial in the staging surgery of type II ECs, omental metastasis was reported as 13% and up to 25% for CCC and SC, respectively, in different studies^(8,9)^.

There is no consensus regarding adjuvant therapy for type II ECs. Adjuvant chemotherapy is usually suggested for patients with type II ECs for all stages, because even patients with uterine SC without myometrial invasion treated with observation alone were shown to have a risk of recurrence, which varied from 0 to 30%^(10)^ and clear cell tumors were reported to have a tendency for distant recurrence^(5)^. Although there are different results in the literature, adjuvant radiotherapy is usually accepted to decrease local recurrence, but an exact effect on survival has not been shown^(3,6)^. In our study, 80% of patients with CCC and 65% of patients with serous tumor received adjuvant therapy (p=0.08).

Recurrence was observed in 22 (27.5%) patients; the recurrence pattern was similar between the 2 groups. Twenty-two percent to 38% of patients were reported to have recurrence in the literature^(11,12,13)^. In the subgroup analysis of patients with CCC, only lymph node involvement was associated with recurrence. In the group with serous tumor, paraaortic lymph node metastasis, presence of LVSI, and ovarian and omental metastasis were associated with a higher risk of recurrence. Additionally, the patients who had recurrence in both groups had higher preoperative Ca-125 levels. Preoperative Ca-125 was also associated with 5-year PFS in patients with CCC in our study (p=0.016). There are no data in the literature on the relation between Ca-125 level and uterine clear cell tumors. On the other hand, the association of uterine SC and Ca-125 level has been studied in several trials. In a study that analyzed the relation between preoperative Ca-125 level and uterine SCs in 41 patients, Olawaiye et al.^(14)^ showed that preoperative Ca-125 was associated with disease stage at diagnosis and with the likelihood of death. In addition, Abramovich et al.^(15)^ reported that a rising Ca-125 was associated with relapse.

In the present study, 5-year PFS was 60.6% and 45.5% for CCC and serous tumor, respectively (p=0.465). These rates were 85.8% and 67.8% for 5-year OS (p=0.565). In the study by Scarfone et al.^(13)^ these rates were reported as 67% and 55% for 5 year PFS, and 77% and 71% for 5-year OS, respectively. Thomas et al.^(16)^ found that 5-year PFS and OS were 46% and 55% in 99 patients with CCC. There is a wide range of survival rates reported in the literature. Different complete staging and cytoreduction rates, and different adjuvant therapies may account for this situation. Complete surgical staging including lymphadenectomy and cytoreduction are recommended to be performed for all patients with these tumors, because more than half of these patients are upstaged during these procedures and residual disease was shown to be associated with worse survival^(6,7)^. Ninety-three percent of our patients underwent complete surgical staging and cytoreductive surgery and the numbers of lymph nodes removed were quite high. These may be the reasons for the high survival rates in our study.

In the current study, in the univariate analysis of the clear cell subgroup, omental metastasis, paraaortic involvement, peritoneal cytology positivity, and receiving no adjuvant therapy were associated with worse 5-year OS, and omental metastasis, paraaortic involvement, pelvic involvement, peritoneal cytology positivity, preoperative Ca-125 level, and ovarian metastasis were associated with 5-year PFS. However, in the study by Thomas et al.^(16)^ age more than 60 years, LVSI, and myometrial invasion equal to or greater than half were reported associated with both 5-year PFS and OS in the univariate analysis that included 99 patients with uterine CCC. In the same study, a multivariate analysis revealed that stage and adjuvant radiotherapy were independent prognostic factors for both PFS and OS, and systemic lymphadenectomy was an independent prognostic factor for OS only. Abeler and Kjørstad^(17)^ showed in a study of 97 patients with uterine CCC that 17% of patients with LVSI survived 5 years, in contrast to 49% of patients without this finding. In the univariate analysis of the serous subgroup of our study population, lower number of harvested lymph nodes, paraaortic metastasis, and LVSI were associated with worse 5-year OS, and paraaortic metastasis, omental metastasis, and LVSI were associated with worse 5-year PFS. Similar findings were shown in a study that analyzed 129 patients with uterine SC. In that study, worse 5-year OS was reported associated with LVSI and positive lymph nodes in the univariate analysis. Different to other studies, the same study also showed that myometrial invasion was related to OS^(18)^. Myometrial invasion was also reported associated with PFS in 83 women with stage I uterine SC. The authors could not show a relation between LVSI, number of harvested lymph nodes, age, and survival^(11)^. Similarly, Fader et al.^(19)^ were unable to demonstrate an association between LVSI and PFS.

### Study Limitations

The retrospective nature of the study was one of our limitations. Additionally, the adjuvant chemotherapy and radiotherapy protocols could not be standardized during the study period. However, this study included 100 patients with CCC and SC ECs from a single institution and the clinico-pathologic features of the patients could be obtained in detail. Most patients in this study underwent complete staging and cytoreductive surgery including comprehensive lymphadenectomy. These are considered to be the advantages of our study.

## CONCLUSION

In this study, with the exception that SC ECs presented at more advanced stages, we could not show a statistically significant difference between patients with CCC and SC ECs regarding surgico-pathologic features, recurrence rates and patterns, and survival rates. However, patients with SC ECs had a tendency to have less favorable characteristics compared with CCC. We also demonstrated factors that affected recurrence and survival.

## Figures and Tables

**Table 1:**
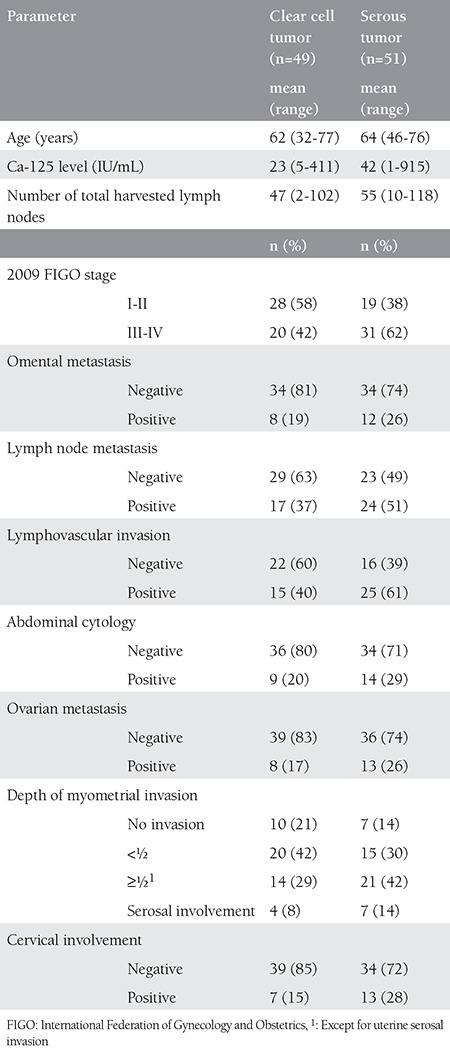
Comparison of clinico-pathologic characteristics of patients

**Table 2:**
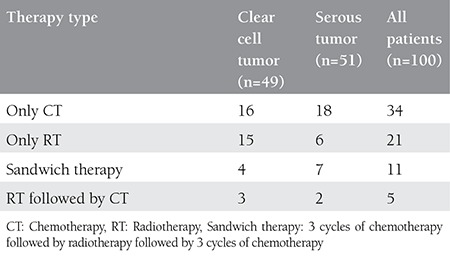
Comparison of adjuvant therapies between the tumor types

**Table 3:**
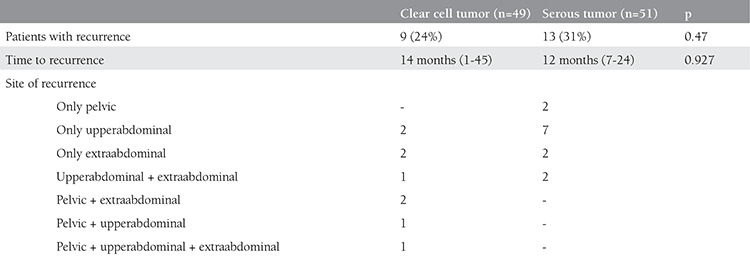
Pattern of recurrence

**Table 4:**
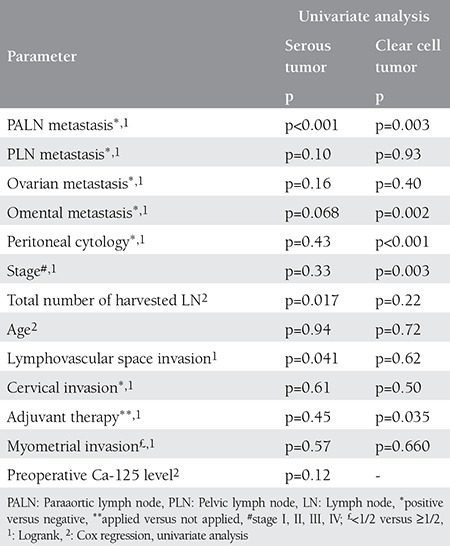
Five-year overall survival

**Table 5:**
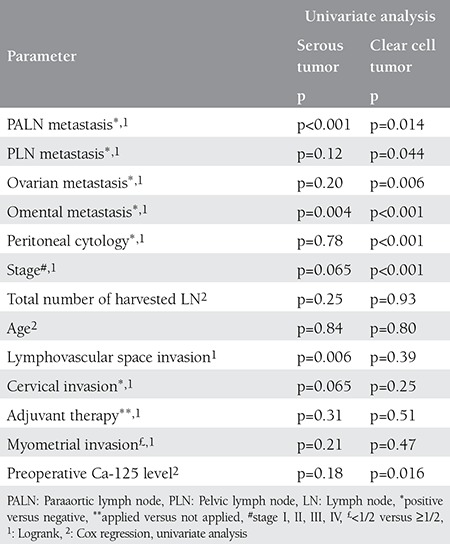
Five-year progression-free survival
